# Enhanced virome sequencing using targeted sequence capture

**DOI:** 10.1101/gr.191049.115

**Published:** 2015-12

**Authors:** Todd N. Wylie, Kristine M. Wylie, Brandi N. Herter, Gregory A. Storch

**Affiliations:** 1 The Department of Pediatrics, Washington University School of Medicine, St. Louis, Missouri 63110, USA;; 2 McDonnell Genome Institute, Washington University School of Medicine, St. Louis, Missouri 63108, USA

## Abstract

Metagenomic shotgun sequencing (MSS) is an important tool for characterizing viral populations. It is culture independent, requires no a priori knowledge of the viruses in the sample, and may provide useful genomic information. However, MSS can lack sensitivity and may yield insufficient data for detailed analysis. We have created a targeted sequence capture panel, ViroCap, designed to enrich nucleic acid from DNA and RNA viruses from 34 families that infect vertebrate hosts. A computational approach condensed ∼1 billion bp of viral reference sequence into <200 million bp of unique, representative sequence suitable for targeted sequence capture. We compared the effectiveness of detecting viruses in standard MSS versus MSS following targeted sequence capture. First, we analyzed two sets of samples, one derived from samples submitted to a diagnostic virology laboratory and one derived from samples collected in a study of fever in children. We detected 14 and 18 viruses in the two sets, comprising 19 genera from 10 families, with dramatic enhancement of genome representation following capture enrichment. The median fold-increases in percentage viral reads post-capture were 674 and 296. Median breadth of coverage increased from 2.1% to 83.2% post-capture in the first set and from 2.0% to 75.6% in the second set. Next, we analyzed samples containing a set of diverse anellovirus sequences and demonstrated that ViroCap could be used to detect viral sequences with up to 58% variation from the references used to select capture probes. ViroCap substantially enhances MSS for a comprehensive set of viruses and has utility for research and clinical applications.

High-throughput, massively parallel nucleotide sequence analysis has made in-depth studies of the human microbiome feasible. Thus far, most microbiome studies have focused on bacteria (Turnbaugh et al. 2009; Arumugam et al. 2011; Gajer et al. 2012; Human Microbiome Project Consortium 2012), although some include fungi (Paulino et al. 2006; Findley et al. 2013; Cleland et al. 2014; Willger et al. 2014) and viruses (Reyes et al. 2010; Minot et al. 2011; Wylie et al. 2012, 2014; De Vlaminck et al. 2013). Viruses are particularly understudied, in part due to the challenges of assessing their presence in clinical samples. Viruses as a group have highly variable genomes, with no gene shared among all viruses that can be surveyed by an amplicon-based sequencing strategy. Therefore, studies of viruses based on nucleotide sequencing require a metagenomic approach. Metagenomic shotgun sequencing (MSS) is a relatively unbiased, culture-independent method in which nucleic acid extracted from a sample is sequenced. Sequence reads are classified based on similarity to reference genomes. This approach allows comprehensive study of the viral component of the microbiome (the virome) and has led to the discovery of novel viruses (for review, see Chiu 2013) and the characterization of viruses present in healthy and sick people (Reyes et al. 2010; Minot et al. 2011; Lysholm et al. 2012; Wylie et al. 2012, 2014; Holtz et al. 2014; Oh et al. 2014; Young et al. 2014). When adequate numbers of sequence reads are generated, viruses can be characterized with regard to taxonomy and the presence of genes associated with virulence and resistance to antiviral drugs.

A limitation of MSS as employed to date for virus detection is that the amount and proportion of viral nucleic acid in samples from humans may be very low, and in these cases, few viral sequences are generated. In our experience using MSS, we have detected fewer than 10 viral sequences per 25 million sequence reads generated for a virus that was detected in a sample by a molecular assay (Wylie et al. 2012). In other instances, we have failed to detect viruses known to be present based on molecular assays (Wylie et al. 2012). These difficulties may reflect the small genome size of some viruses and/or low levels of virus in the sample. This can be a particular problem for studies of the virome of healthy, asymptomatic individuals (Wylie et al. 2012, 2014), in whom virus levels may be low. In efforts to increase the sequence yield, purification or enrichment procedures have been employed, including low-speed centrifugation and/or filtration to remove bacterial and host cells, sample treatment with nucleases to digest nucleic acid not protected within virions (Allander et al. 2001), or concentration of viral particles by high-speed gradient centrifugation (for review, see Duhaime and Sullivan 2012). Each of these procedures may bias against detection of some viruses (Breitbart and Rohwer 2005; Young et al. 2014).

An alternative method for enrichment of viral sequences in a metagenomic sample prior to sequencing is targeted sequence capture, a well-established approach for targeted enrichment of specific nucleic acids. Targeted sequence capture has been used extensively to assess the human exome, as well as specific gene targets (Lovett et al. 1991; Albert et al. 2007; Hodges et al. 2007; Okou et al. 2007). Sequence capture has also been applied to the study of specific viruses (Depledge et al. 2011; Duncavage et al. 2011; Koehler et al. 2014). Our aim was to develop a comprehensive viral targeted sequence capture panel that could be used to (1) assess all viruses known to infect vertebrate cells and (2) detect divergent viruses. To this end, we created ViroCap, a targeted sequence capture panel that enhances the detection of a comprehensive set of viruses with vertebrate hosts. Here we describe the first application of ViroCap to enrich a broad range of viruses from human clinical samples.

## Results

**Figure 1.**
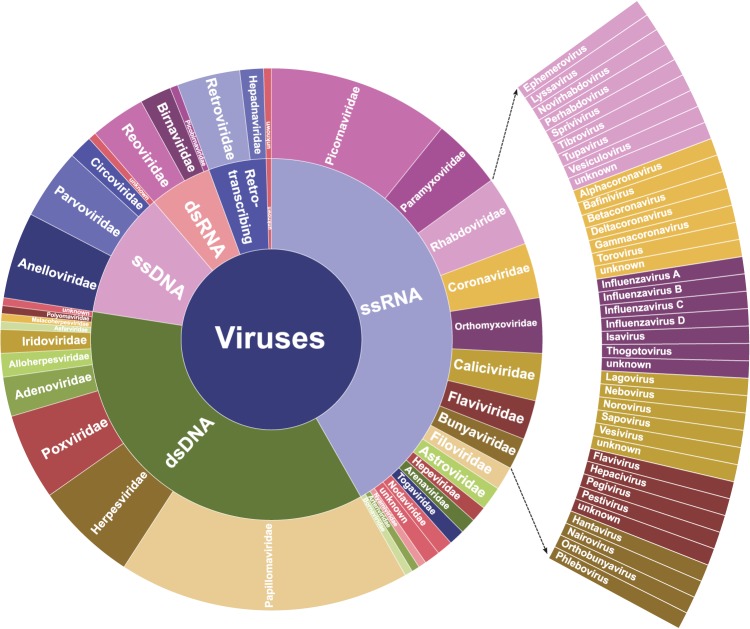
Taxonomic distribution of target genomes included in ViroCap. Shown are the viral groups and families included in the ViroCap targeted sequence capture panel. A highlighted subset illustrates underlying genera. To view complete genera for all families, see Supplemental Figure S1A. Taxonomic assignments were obtained from the NCBI Taxonomy Viewer (http://www.ncbi.nlm.nih.gov/genomes/GenomesGroup.cgi?opt=virus&taxid=10239).

ViroCap includes targets from 34 viral families, comprising 190 annotated viral genera and 337 species (Fig. 1). Included viruses represent all DNA and RNA viruses with sequenced genomes from vertebrate hosts, except human endogenous retroviruses, which were excluded due to their prevalence within the human genome. Nearly 1 billion bp of viral genome sequences were condensed into <200 million bp of targets (Supplemental Table S1) using *k*-mer and clustering analyses to define a unique set of reference sequences, as described in the Methods.

### Analysis of clinical and research samples with ViroCap

We evaluated the effectiveness of detecting DNA and RNA viruses in MSS data compared with ViroCap targeted sequence capture data in two sets of human samples. In experiment 1, the sample set consisted of clinical samples that had been found to be positive by molecular tests in the Diagnostic Virology Laboratory at St. Louis Children's Hospital. Nucleic acid extracts available in the Virology Laboratory were pooled, and a sequencing library was prepared from this pooled nucleic acid (see Methods). In experiment 2, eight patient samples from a research study of young children with fever (Colvin et al. 2012; Wylie et al. 2012) were selected for use in the present study because each had been found to be positive for one or more viruses when tested by batteries of PCR assays used in that study. Individual sequencing libraries were prepared from each of the eight samples as described in the Methods and pooled for sequencing. Experiments 1 and 2 were analyzed in separate sequencing runs. In each experiment, sequencing libraries were divided, and the same library was sequenced without targeted sequence capture (precapture) and following targeted sequence capture using ViroCap (post-capture).

**Figure 2.**
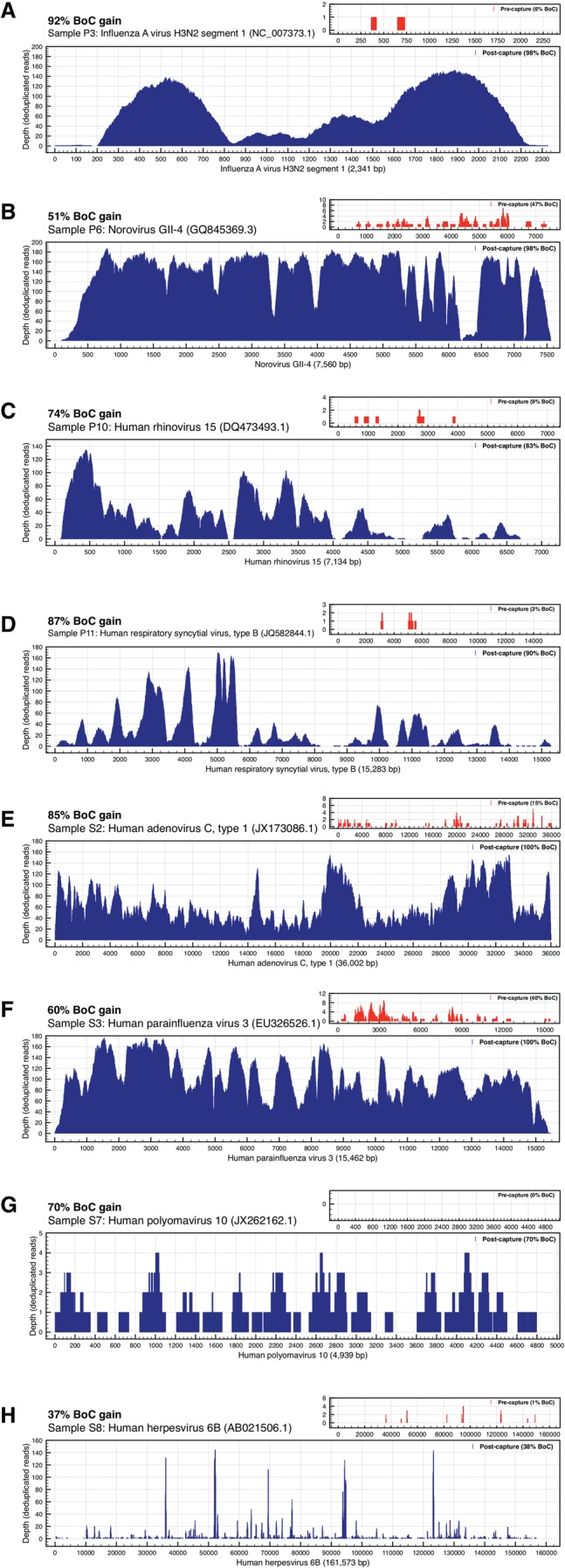
Targeted sequence capture enrichment. Examples are given showing the impact of targeted sequence capture on breadth and depth of genome coverage for eight representative viral genomes (*A*–*H*). For illustrative purposes, all of the coverage panels in this figure have been normalized by removing (*deduplicating*) reads based on identical alignment start-sites. Nucleotide positions along the reference genome are shown on the *x*-axis. The depth of deduplicated reads is shown on the *y*-axis. The shaded portion indicates the sequence coverage (breadth and depth) for each virus. Post-capture sequence coverage is represented in the larger panels in blue; precapture sequence coverage is shown in the *insets* in red. Note that *y*-axis ranges are different for each panel. At the *top* of each panel is shown the breadth of coverage (BoC) for the sample. The header of each panel includes breadth of coverage gain (BoC gain), sample id, and reference genome name and NCBI version number. BoC gain is calculated by subtracting the percentage of the length of the reference genome that was covered by sequence reads in precapture MSS from the percentage of the length of the reference genome covered by post-capture sequence reads.

In experiment 1, we detected 10 viruses in the precapture MSS data (Table 1). After targeted sequence capture using the same sequencing library, we detected the same 10 viruses plus four additional viruses. Targeted sequence capture resulted in dramatic improvements in all sequence coverage metrics (Table 1; Supplemental Table S2), including number and percentage of viral reads, breadth and depth of coverage, and coverage gaps. In experiment 1, the median increase in percentage of viral reads was 674 (range, >13–9335), and the median breadth of coverage increased from 2.1% (range, 0%–89.8%) to 83.2% (range, 0.8%–100%). Illustrative examples are shown in Figure 2A–D.

**Table 1.**
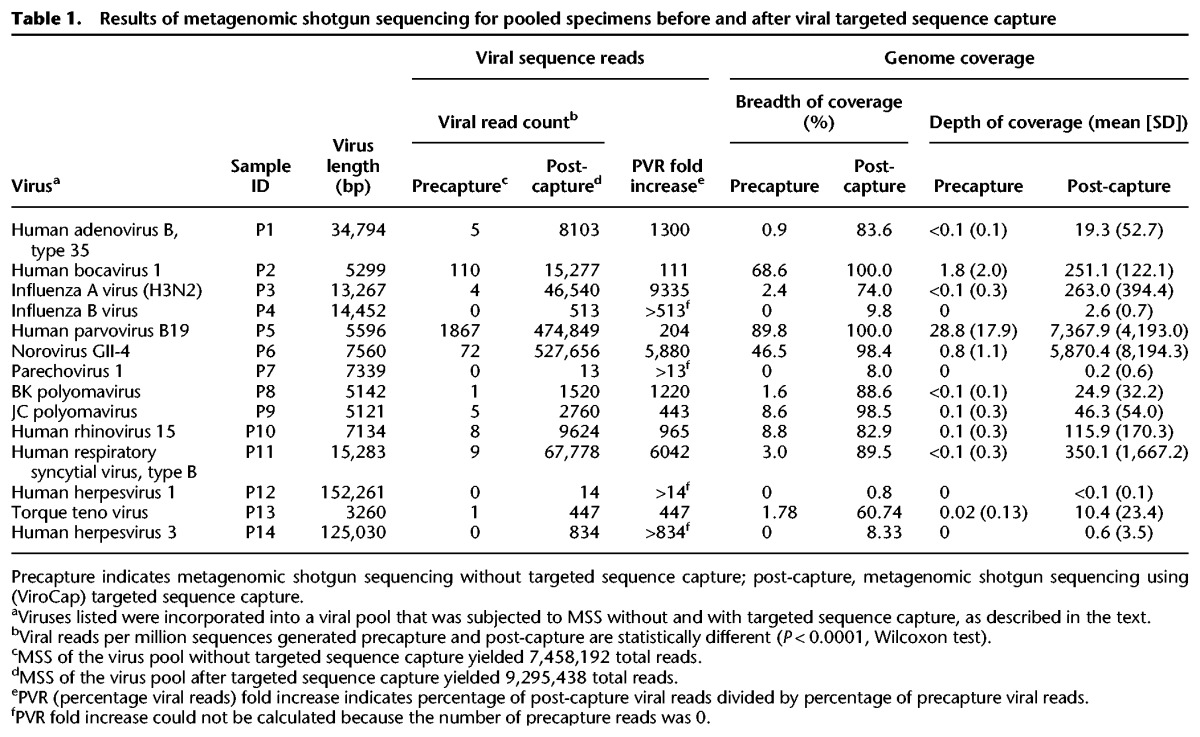
Results of metagenomic shotgun sequencing for pooled specimens before and after viral targeted sequence capture

In experiment 2, 11 viruses were detected in the precapture MSS data (Table 2). After targeted sequence capture with ViroCap using the same sequencing libraries, we detected those 11 viruses plus seven additional viruses. Thus, in the two experiments together, the number of viruses detected went from 21 to 32, a 52% increase. All of the viruses detected in both experiments were confirmed by PCR assays except for a torque teno virus in the clinical pool, which was not evaluated by PCR (Table 1; Supplemental Tables S8, S9). Viruses detected encompassed 19 genera from 10 families (Supplemental Fig. S1). In experiment 2, we again found that targeted sequence capture resulted in dramatic improvements in sequencing parameters. In experiment 2, the median fold increase in percentage of viral reads was 296 (range, >56–2722), and the median breadth of coverage increased from 2.0% (range, 0%–99.9%) to 75.6% (range, 13.5%–100%). Illustrative examples are shown in Figure 2E–H.

**Table 2.**
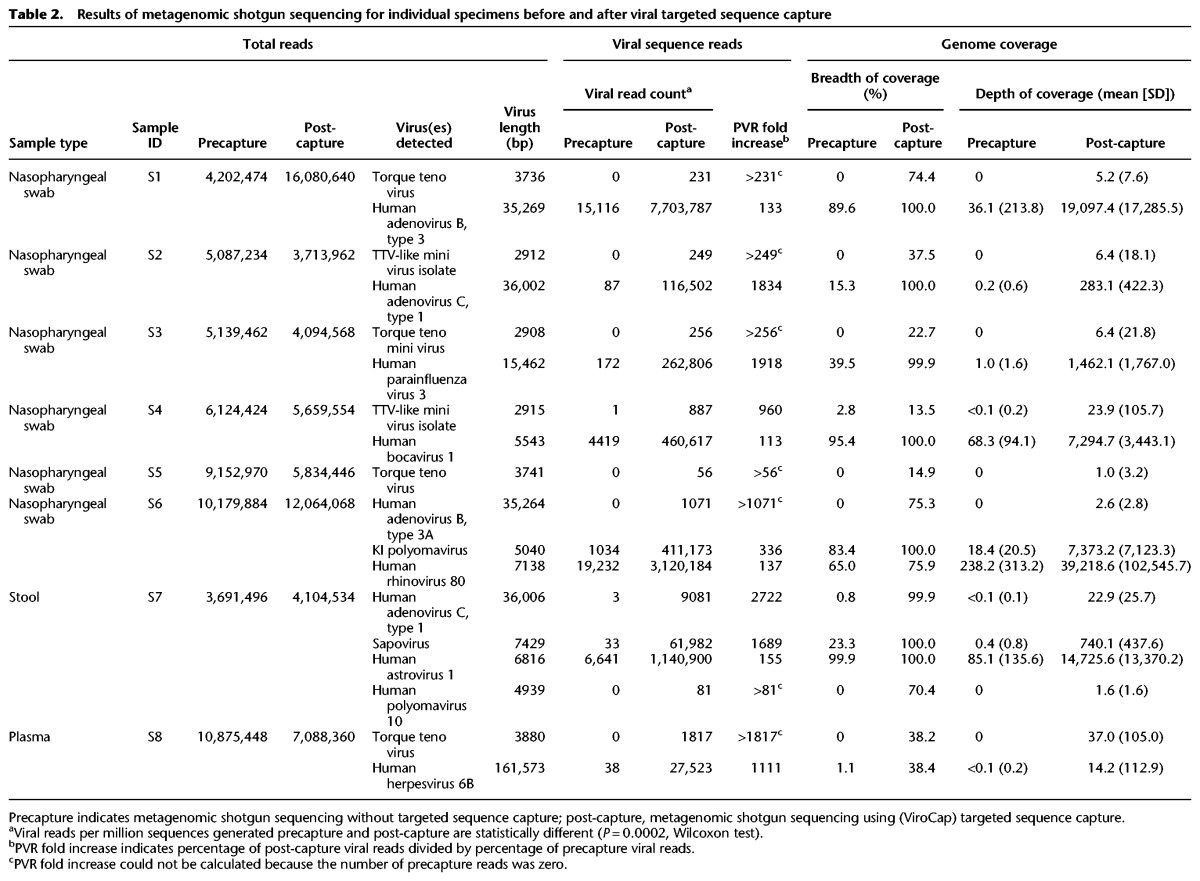
Results of metagenomic shotgun sequencing for individual specimens before and after viral targeted sequence capture

By use of targeted sequence capture, >80% breadth of coverage of the viral genomes was obtained for 16 of 32 viruses, including diverse DNA and RNA genomes of sizes ranging from 5–161 kb (Tables 1, 2; Supplemental Tables S2, S3). Greater than 90% breadth of coverage was obtained for 12 of 32 viruses, and eight viruses had 100% coverage. Precapture, the median gap size in genome coverage was 1704 bp (range 4–152,261 bp), and post-capture, the median gap size was 82 bp (range 0–13,734 bp) (Supplemental Tables S2, S3). High genome representation was obtained for multiple viruses in the same capture reaction, as experiments 1 and 2 were each single, independent capture reactions encompassing multiple samples (see Methods).

### Targeted sequence capture identifies divergent viral sequences

**Figure 3.**
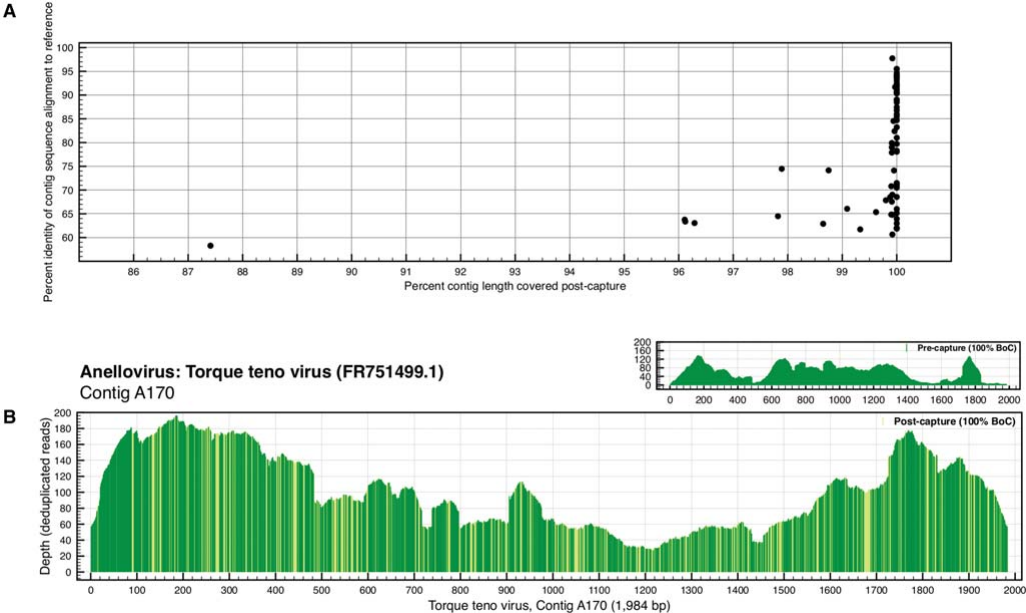
Targeted sequence capture identifies divergent sequences. (*A*) The percentage identity of the top high-scoring segment pair (HSP) identified from the BLAST alignment of anellovirus contig sequences to the references used to design ViroCap is plotted on the *y*-axis. The *x*-axis represents the percentage of the length of the anellovirus contig covered after targeted sequence capture. (*B*) This coverage plot represents the sequence coverage of a divergent anellovirus contig sequence. The figure is designed as described in the figure legend for Figure 2, with the following addition: The post-capture coverage plot is shaded to show regions of nucleotide sequence variation between the anellovirus contig and the most similar reference genome in the ViroCap panel. Dark shading represents areas of identical sequence, and each position with nucleotide mismatch between aligned sequences is shown in the lighter color. All of the HSPs are shown, rather than just the top HSP.

To determine whether or not divergent sequences could be identified using targeted sequence capture, we tested ViroCap on samples containing anelloviruses, a highly divergent group of ssDNA viruses that have a common genome structure but may have up to 30%–50% nucleotide sequence diversity among separate species (Ninomiya et al. 2007; de Villiers et al. 2011). We selected anellovirus-positive samples that we had previously characterized using multistrand displacement amplification followed by high-throughput sequencing. After assembling the precapture sequences to generate contiguous sequences (contigs), we identified anellovirus contigs >1 kb in length. The contigs had varying degrees of similarity to the reference genomes used in the ViroCap panel based on BLAST alignments, ranging from 58%–98% nucleotide sequence identity for the top high-scoring segment pair (HSP) alignment (Fig. 3A; Supplemental Table S4). All of the contigs assembled using the precapture sequence data were also detected post-capture. The contig with 58% identity to the reference database was missing 13% of its length post-capture (Fig. 3A). The contig with the next lowest percentage identity to the reference database (62%) was fully sequenced (i.e., 100% breadth-of-coverage) (Fig. 3A,B). Figure 3B illustrates the nucleotide sequence matches/mismatches between the contig and the most similar reference genome in the sequences used for the ViroCap design. These results demonstrate that targeted sequence capture using the ViroCap panel allows us to identify variant virus sequences having as low as 58% nucleotide sequence identity.

### Specificity of targeted sequence capture

In order to determine whether ViroCap systematically enriched off-target sequences, we compared the filtering and classification statistics of the nonviral sequences in the precapture MSS and targeted sequence capture data (Supplemental Table S5; Supplemental Figs. S2, S3). If our probes were specific, we would not observe any systematic enrichment of specific human chromosomes or bacterial genomes post-capture. However, we anticipated a small amount of variation because the targeted sequence capture library had been through more sample handling in the form of incubations, dilutions, and amplifications. We found that the proportions of the nonviral sequences were strongly correlated (Pearson's correlation value: *r* = 0.9881–0.9996) (Supplemental Table S5; Supplemental Fig. S2). A slightly higher percentage (mean, 5.8%; median, 5%; range, 0%–10.7%) of reads aligned to nonviral reference genomes in the post-capture data compared with precapture in all but one of the samples. However, the distribution of sequences among reference genomes did not show a systematic bias. This can be seen in the conserved distribution of sequences among human chromosomes (Supplemental Fig. S3).

## Discussion

We designed the ViroCap panel to enhance the sensitivity of MSS for comprehensive detection of known vertebrate viruses, as well as to detect divergent viruses that have nucleotide sequence similarities to known viruses. Here we have demonstrated that targeted sequence capture using ViroCap dramatically increases the amount of viral sequence obtained from human samples compared with conventional MSS, greatly enhancing the resolution of genomic characterization and increasing the number of viruses detected by >50%. Enhancement was demonstrated for DNA and RNA viruses from multiple diverse families. The increased sensitivity will be valuable in multiple research applications, including descriptions of the human virome, and will also improve the potential for MSS as a diagnostic tool in human and animal health.

The dramatic enrichment of viral nucleic acids present within the targeted sequence capture libraries offers important advantages. First, as we demonstrate, MSS with ViroCap can be used to generate complete or nearly complete genome sequences directly from clinical samples, including those with very low proportions of viral nucleic acid, without culturing the viruses. Availability of extensive sequence data provides the opportunity to distinguish among closely related virus subtypes or even among viral strains, which might not be distinguished by other types of assays. In the data set presented here, we demonstrated the ability to type rhinoviruses and distinguish between human herpesvirus 6B and 6A, adenovirus types A and C, and polyomaviruses JC and BK. Notably, influenza A virus was identified precapture but could only be typed as an H3N2 virus post-capture. Elsewhere, we used ViroCap to sequence the enterovirus D68 genome directly from clinical samples (Wylie et al. 2015), and in that work, the extensive sequence data that we obtained allowed us carry out detailed comparative analysis of closely related strains that differed at a limited number of nucleotide positions. Second, the use of ViroCap can reduce the depth of sequencing needed to detect viruses in samples. Because targeted sequence capture results in a large increase in the percentage of sequencing reads that are viral (Tables 1, 2; Fig. 2; Supplemental Tables S2–S4), ViroCap achieves better viral coverage while requiring the generation of fewer total sequence reads. This increased efficiency has the potential to lower sequencing costs.

An important feature of ViroCap is the tiling of capture probes across genomes, including highly conserved regions that may allow detection of genomic fragments of divergent viruses that share little overall sequence homology with known viruses. We illustrated such capability using anelloviruses containing divergent nucleotide sequence (Fig. 3). In addition, the inclusion of Genome Neighbor targets enhanced our design not only by expanding beyond the tiled Reference Sequence (RefSeq) viruses but also by adding sensitivity for genomic regions where RefSeq capture probes alone might not have captured divergent strains (see Methods). ViroCap cannot detect viruses that do not share any nucleotide sequence similarity to known viruses; however, we note that because the enrichment of viral nucleic acids occurs after sequence library construction, the uncaptured portion of the library could subsequently be sequenced for additional attempts at pathogen discovery. Furthermore, the ViroCap panel is extensible and will be updated periodically with new viral sequences as they are added to RefSeq and the Genome Neighbors databases. Updates will be publicly available through our GitHub repository (see Data Access).

There were a few genomes (fewer than 10) in the NCBI reference databases that had been cloned into bacterial vectors prior to sequencing, and the deposited viral genome sequences contained bacterial vector sequence. We were not aware of this prior to probe design, so ViroCap includes capture probes that target these sequences. This resulted in enrichment of some sequences (on average 1.1% of total nonviral reads) that were subsequently recognized by our analysis pipeline as bacterial based on nucleotide sequence alignment. In subsequent versions of ViroCap, we will filter out these bacterial vector sequences.

In the experiments reported here, we pooled sequencing libraries prior to targeted sequence capture in order to reduce cost, but we still achieved enhanced detection of multiple viruses of varying abundance. As has been reported for strategies that involve sequencing indexed, pooled libraries (Kircher et al. 2012), we observed some sample cross-contamination. This cross-contamination is recognizable when a high number of viral sequences are detected in the truly positive sample, while few sequences (<0.05% of the viral sequences in the truly positive sample) of the same virus are detected in other samples in the pool. In a clinical setting, each sample would optimally be captured and sequenced independently to reduce the possibility of sample cross-contamination. However, future methodological improvements could allow pooling of clinical specimens.

The success of viral targeted sequence capture is affected by the representation of the virus in the sequencing library. In our sample preparation, total nucleic acid extracted from the sample was reverse transcribed and randomly amplified prior to library construction (Wang et al. 2003), allowing detection of DNA and RNA viral genomes within the same sequencing experiment. The uneven sequence representation observed for some genomes (Fig. 2) is likely due in part to detection of messenger RNA, whose abundance reflects patterns of gene expression, as well as to primer biases during the reverse transcription and amplification steps. Capture hybridization may also induce bias, in that sequences that diverge from target probe sequences may be captured less efficiently than those with exact or nearly exact matches to the probe. Taken together, these data suggest that further improvement in the performance of viral targeted sequence capture may be achievable by improving efficiency of reverse transcription, amplification, and library construction, while continuing to update the ViroCap panel as new, divergent genome sequences become available.

Methods other than genome sequencing have been used for virus characterization and discovery, including Virochip, a microarray-based method for detection/genotyping of viral pathogens (Wang et al. 2002; Chen et al. 2011), and PathoChip, a microarray designed to detect viruses and other microbial pathogens (Baldwin et al. 2014). While designed to detect known viruses by means of microarray probe spotting, this technology has also shown the ability to detect emerging viruses (Wang et al. 2003; Yu et al. 2012). The primary difference between the designs of these microarrays and ViroCap targeted sequence capture is that the latter approach targets complete viral genomes while the microarrays target smaller, discrete genomic regions. The results obtained from each approach also differ significantly. The microarray approach detects the presence of a virus but does not directly provide sequence information. In contrast, MSS enhanced by ViroCap targeted sequence capture provides sequence data, sometimes covering the entire genome.

In conclusion, ViroCap greatly enhances the sensitivity of MSS for nucleotide sequence–based virus detection. To our knowledge, ViroCap represents the first effort to apply a targeted sequence capture approach to the detection of a comprehensive set of viruses. Its research applications are far reaching, allowing a new, higher-resolution view of eukaryotic DNA and RNA viruses in the microbiome. ViroCap should also help realize the potential of MSS as a clinical diagnostic tool that can simultaneously detect viruses and provide immediate characterization, including taxonomic assignment, strain typing, virulence characteristics, and anti-viral drug resistance genotyping. ViroCap could also be modified into a tool for broader pathogen identification, which might include a comprehensive set of human pathogens: genes from viruses, bacteria (e.g., toxin genes, antibiotic resistance genes), fungi, protists, and other microbes.

## Methods

### Taxonomy selection

At the time, we designed the ViroCap panel, NCBI GenBank had available for download a total of ∼1 Gb of sequence representing 440 viral families, well beyond the 200 Mb of target space supported by the custom SeqCap EZ library format (NimbleGen). Therefore, we developed the following approach for selecting representative targeted sequence capture probes. Because we were interested in studying viral diseases of humans, we excluded bacteriophages and endogenous human retroviruses. We also specifically did not include references from the following NCBI viral reference genome database host categories: algae, archaea, bacteria, diatom, environment, fungi, invertebrates, plants, and protozoa. After filtering, our target list contained reference sequences from the following host categories: human, vertebrates, and “unknown.” This list included viruses that could have both vertebrate and invertebrate hosts, such as vertebrate viruses with insect vectors. Based on these broad viral-host categories, we downloaded all of the associated viral reference sequences in each chosen category from NCBI (accessed February 3, 2014). These sequences comprise the core reference database from which our capture library is designed. Our capture library includes targets from 34 viral families composed of 190 annotated viral genera and 337 species (Fig. 1; Supplemental Tables S6, S7). Sources of viral sequences include complete representation of the viral genomes from NCBI's RefSeq collection, complementary representation of unique regions from Genome Neighbor targets, selected representation of NCBI Influenza Virus Resource sequences, and the entirety of the probe space represented on the Virochip microarray (Yu et al. 2012), GEO accession number GPL15905. The methods used to consolidate these database sequences follow.

### RefSeq

NCBI's RefSeq (http://www.ncbi.nlm.nih.gov/refseq/) genome collection is a database of taxonomically diverse entries representing comprehensive, well-annotated genome sequences (Pruitt et al. 2014; Tatusova et al. 2014). As RefSeq entries are the most complete sequence representatives in terms of annotation and metadata consistency, we targeted selected viral RefSeqs by tiling of targeted sequence capture probes across the entire length of each RefSeq's genome, with the intention of capturing the entire viral genome. For our capture library, RefSeq nucleotide FASTA sequences were downloaded for desired viral-host categories (human; vertebrates; vertebrates, human; vertebrates, invertebrates; vertebrates, invertebrates, human; invertebrates, vertebrates; unknown) using both the online NCBI taxonomy viewer (http://www.ncbi.nlm.nih.gov/genomes/GenomesGroup.cgi?opt=virus&taxid=10239), as well as the RefSeq-specific FTP site (ftp://ftp.ncbi.nlm.nih.gov/refseq/release/viral). Entries were merged to avoid redundancy. RefSeq targets were pooled with the other sequence candidates (see Design Consolidation). A total of 1456 RefSeq FASTA entries (26.9 Mb) representing 190 viral genera were completely tiled for inclusion in the ViroCap library, accounting for 13.5% of the total capture library's target space.

### Genome Neighbors

While RefSeq entries are single, canonical species representations, other complete or partial viral sequences also exist in DDBJ/EMBL/GenBank. In the case of viral sequences, there is extensive redundancy in these databases due to the large number of similar viral strains, isolates, and mutants. Therefore, non-RefSeq (e.g., DDBJ, EMBL, GenBank) nucleotide sequences of complete viral genomes that belong to the same species as a RefSeq sequence are classified as Genome Neighbors for that reference sequence, provided that they match all of the criteria that were used to select complete genomic sequences (Bao et al. 2004). At the time of our ViroCap panel design, Genome Neighbors (sequences downloaded from Entrez Genome link “Other genomes for species”; accessed February 3, 2014) in total represented an additional 56,314 entries and 507.1 Mb of sequence, more than 2.5 times our SeqCap EZ capture target sequence space limit. Therefore, an alternative target selection approach was employed to add diversity to our RefSeq selections by selecting unique, complementary Genome Neighbor sequences.

### RefSeq and Genome Neighbor sequence association

We began the process of variant sequence selection by identifying conserved regions in Genome Neighbors already represented by completely tiled RefSeq capture probes. First, we associated our viral RefSeq selections with corresponding Genome Neighbors. This was performed by downloading Genome Neighbor annotation files from NCBI (http://www.ncbi.nlm.nih.gov/genomes/GenomesGroup.cgi?opt=virus&taxid=10239) and associating the information with our RefSeq annotation files, by means of ad hoc Perl parsing and coupling scripts (for results, see Supplemental Tables S6, S7). Once associated, the parent RefSeq sequences could be compared with related Genome Neighbor sequences to determine conserved and divergent nucleotide regions. Each viral RefSeq entry was individually reviewed, along with associated Genome Neighbor entries. FASTA sequences were collected for each RefSeq entry and its related Genome Neighbors for subsequent *k*-mer analysis.

### *K*-mer analysis

Each of the Genome Neighbor sequences was split into 100-bp *k*-mers by means of an exhaustive 1-bp sliding window algorithm, as depicted in Supplemental Figure S4. The resultant output thus included all possible 100-bp sequences based on the combined Genome Neighbor sequence space. As our SeqCap EZ targeted sequence capture probe lengths are 100 bp, the sequences generated by the sliding window algorithm represent the total number of possible probe combinations based on the aggregate of Genome Neighbor sequences. Based on our conservative expectation of hybridization/homology at the capture probe level, we then clustered all of the Genome Neighbor 100-mers back to the parent RefSeq sequence at ≥90% sequence identity using length-sorted FASTA entries and the UCLUST (Edgar 2010) package (version 1.1.579; parameters: –rev –id 0.90). Given that all of our candidate sequences were 100 bp in length and all RefSeq entries are >100 bp, UCLUST always used the longer RefSeq as the first seed (*centroid*) in which to attempt folding of other sequences. As the parent RefSeq had complete probe tiling in our design, any Genome Neighbor 100-mer with ≥90% identity was considered already represented in our capture library and therefore discarded. Genome Neighbor 100-mers with <90% identity were chosen for inclusion in the capture library. As the sliding window approach produces 100-mers that overlapped one another, we merged overlapping 100-mers based on their Genome Neighbor genomic coordinates into single contiguous spans using BEDTools (Quinlan and Hall 2010) functions.

### Genome Neighbor sub-sequences

Resultant subsequences were excised as FASTA entries from corresponding Genome Neighbor references using WU-BLAST's (http://blast.wustl.edu) xdget application and added to the ViroCap panel. These supplementary entries are easily identifiable in our final target design, as the FASTA headers for the entries list the original parent sequence ID with the excised span indicated in curly braces (e.g., gi|1249624|emb|A28090.1| HPV42 [partial] genomic sequence {SQ 2444-2644}). In this manner, for each RefSeq species, we generated Genome Neighbor subsequences from 100 bp to 21 kb in length to add to our capture panel.

These processing steps reduced the aggregate input Genome Neighbors targeted sequence space from 507.1 Mb to 153.2 Mb (Supplemental Table S1), and these sequences were pooled with our other targeted capture sequence targets (see Design Consolidation). A total of 130,808 partial Genome Neighbor FASTA entries (153.2 Mb) were added for capture in our ViroCap library, accounting for 77.1% of the total capture library's target space.

### Influenza Virus Resource

We obtained reference sequences from NCBI's Influenza Virus Resource database (http://www.ncbi.nlm.nih.gov/genomes/FLU/FLU.html), which contains sequence data from the NIAID Influenza Genome Sequencing Project, as well as from GenBank. At the time of our capture panel design, the NCBI Influenza Virus Resource contained 305,524 influenza entries, representing 458.1 Mb of sequence. This is 17 times the size of our viral RefSeq selections and three times the size of our collapsed Genome Neighbor targets. Our selected RefSeq sequences included 29 influenza RefSeq entries (each influenza virus segment is represented as a separate entry), targeted in its entirety. These sequences served as the core of influenza reference genomes against which all other influenza sequences were compared. We directly clustered the long influenza sequences using length-sorted FASTA and the UCLUST package (version 1.1.579; parameters: –rev –id 0.90). In UCLUST, a cluster is defined by one sequence, known as the *centroid* or representative sequence. To lessen the computational burden and ensure that our core influenza RefSeq genomes were always the longest first seeds (*centroids*) in UCLUST's clustering process, we artificially concatenated the 29 parent RefSeq sequences into one linear sequence representation and then split this representation into six segments ranging in size from 18–26 kb. UCLUST preferentially seeded with the long RefSeq construct segments when clustering, ensuring that clustering was first attempted within the longer, canonical references. ULCUST was run with a requirement of ≥90% sequence identity to fold into a parent influenza RefSeq entries segment. Therefore, only sequences that (1) had <90% identity to influenza RefSeq entries and (2) were subsequent centroids in non-RefSeq clusters were chosen for inclusion in our capture panel. This process reduced the aggregate input Influenza Resource Database reference sequence from 458.1 Mb to 15.7 Mb (Supplemental Table S1). Finally, supplementary influenza targets were pooled with the other sequence candidates (see Design Consolidation). A total of 9759 influenza FASTA entries (15.7 Mb) were added for targeted sequence capture in our ViroCap library, accounting for 7.9% of the total capture library's target space.

### Virochip microarray

Considering the biologically important short sequence signatures represented on the Virochip panel (Yu et al. 2012), as well as the comparatively small footprint, we subsumed these sequences within our targeted sequence capture panel design. The probe sequences for the microarray are publicly available at NCBI's Gene Expression Omnibus (GEO) repository (Edgar et al. 2002). We downloaded this information for Platform GPL15905 (Viro5AG-60k) as a text file (http://www.ncbi.nlm.nih.gov/geo/query/acc.cgi?acc=GPL15905). This platform included more than 60,000 oligonucleotides of length 60–70 bp, corresponding to 3.1 Mb of probes (Supplemental Table S1). Virochip targets were pooled with the other sequence candidates (see Design Consolidation). Upon review, 1.3 Mb of the probes were already directly represented by RefSeq, Genome Neighbor, and Influenza Viral Resource sequences during capture library design and synthesis. Therefore, the remaining 25,749 (60–70 bp) Virochip FASTA entries of 1.8-Mb total size were added to ViroCap, accounting for <1% of the total targeted sequence capture panel.

### Design consolidation

All of our selected candidate target sequences from RefSeq, Genome Neighbors, Influenza Virus Resource, and the Virochip microarray were combined into a single FASTA sequence file. Human endogenous retroviruses were removed from inclusion by means of a two-part process: (1) Entries were filtered by taxonomic annotation indicating human endogenous retrovirus identity, and (2) the remaining entries were BLAST-aligned to the GRCh37-lite build of the human reference genome to remove sequences with high percentage identity (≥75%) at the 100-bp probe level. Finally, sequences were hard-masked (i.e., bases converted to N's) in low complexity regions using the DUST (R Tatusov and DJ Lipman, unpubl.) software module. The final ViroCap targeted sequence capture panel consists of 185,835 FASTA sequences totaling 198.9 Mb (see Taxonomy Selection).

### NimbleGen sequence capture design

Our consolidated target sequences were submitted to Roche NimbleGen on March 31, 2014 for capture library design and synthesis. As our final ViroCap design required 198.9 Mb, manufacturing was implemented under the custom NimbleGen SeqCap EZ Developer Library format, which has a maximum capture space of 200 Mb of nonhuman sequence. NimbleGen's Sequence Capture design offered up to 2.1 million of 50–105mer sequence probes. It was at the discretion of NimbleGen, based on proprietary algorithms, to redistribute probes for better capture uniformity, redundancy, and comprehensive target base coverage. NimbleGen provided us with a proposed capture design accompanied by coordinate (GFF, BED) files and associated sequence coverage metrics on April 14, 2014. The design set contained probe representation generated by first masking all but one exact copy of each 100-mer in our original FASTA file, tiling the unmasked regions, screening the resulting probes against the (hg19) human genome, and finally selecting only those probes that had no matches in the human genome as determined by the SSAHA (Ning et al. 2001) algorithm. NimbleGen provides two metrics for assessing in silico targeted sequence capture design coverage: (1) Target bases covered with 0-bp-offset are determined by counting target bases directly represented in probe sequences, and (2) target bases covered with 100-bp-offset are determined by counting all target bases within 100 bp of a probe. The capture design provided 95.9% 0-bp-offset coverage and 99.6% 100-bp-offset coverage of our initial 198.9-Mb target request. We approved the design on April 17, 2014 for capture library synthesis and received our first 12 SeqCap EZ Library reactions for in-house Illumina sequencing and analysis on April 28, 2014.

### Human subjects approval and sample selection

Samples were collected under protocols approved by the Human Research Protection Office at Washington University School of Medicine (IRB protocol nos. 201106177, 201102561, and 201102045). Samples were selected to represent a broad range of viruses that are commonly encountered in the clinical laboratory and in our research studies. Viruses were identified in samples based on clinical laboratory test results in the Diagnostic Virology Laboratory at St. Louis Children's Hospital or by PCR assays and sequencing results carried out in previous studies (Colvin et al. 2012; McElvania TeKippe et al. 2012; Wylie et al. 2012). Specimens of nasopharyngeal secretions, plasma, and stool were included.

### Sequencing

Total nucleic acid was extracted from clinical samples using the EasyMag NucliSENS instrument (bioMerieux). Samples were processed in one of two ways. In experiment 1, nucleic acids from clinical specimens from the Diagnostic Virology Laboratory were combined, and the resulting pooled nucleic acid was used as input for a single sequencing library (constructed as described below). These samples are designated with a sample identification prefix of “P” in the various tables and figures. Alternatively, in experiment 2, individual sequencing libraries were made from each set of eight different specimens prior to combining the libraries for sequencing. These samples are designated with a sample identification prefix of “S” in the various tables.

For sequencing libraries, DNA and RNA viruses were assessed in the same assay as previously described (Wang et al. 2003). Specifically, the RNA in the total nucleic acid was reverse transcribed with reverse transcriptase (Promega) and random nonomers tagged with a conserved sequence (5′-GTTTCCCAGTCACGATA-3′) to be used for subsequent amplification (Integrated DNA Technologies), and second strand synthesis was carried out with Sequenase V2.0 DNA polymerase (Affymetrix). DNA and RNA were subsequently amplified with Accuprime Taq (Life Technologies) using the conserved sequence on the ends of the random primers, and the DNA/cDNA mixture was sheared using the Qsonica Q800R instrument (Qsonica) to generate fragments with an average length of 500 bp. Dual-indexed sequencing libraries were constructed using the KAPA low throughput library construction kit (KAPA Biosystems).

For the anellovirus samples, DNA was amplified with the Illustra GenomiPhi V2 DNA amplification kit (GE Healthcare Life Sciences); RNA was not assessed. DNA was sheared, and libraries were constructed from each sample as described above. Sequencing libraries were pooled.

In each case, the libraries were divided, and part was directly sequenced (precapture) and part was subjected to targeted sequence capture with the custom ViroCap probes prior to sequencing (post-capture). Targeted sequence capture was carried out according to the manufacturer's specifications. We carried out 10, 10, and 16 cycles of post-capture linker—mediated PCR for experiments 1 (pooled clinical samples), 2 (individual samples from the research study), and 3 (anellovirus samples), respectively, prior to sequencing. The number of cycles was empirically determined to be the minimum number needed to obtain a 5 nM dilution of library material for qPCR and loading. Libraries were sequenced on the Illumina HiSeq 2000 or HiSeq 2500 instrument, generating 100-bp paired-end reads.

### Sequence analysis

Viral sequences were identified based on nucleotide and translated protein sequence alignment against reference genomes. The pipeline is adapted from previously described methods (Wylie et al. 2014), except that nucleotide alignments were carried out using BWA-MEM with default settings (Li and Durbin 2009). Because many similar genomes are included in the reference database, we used the initial alignment statistics for each sample to choose a single reference from each species to calculate and report coverage statistics. References were chosen based on having the highest number of reference bases covered. Sequences were realigned to the selected references with BWA-MEM for calculation of coverage statistics and comparison of samples precapture and post-capture. Sequence alignments were evaluated with SAMtools (Li 2011), and sequence coverage was determined with RefCov (http://gmt.genome.wustl.edu/packages/refcov/) and visualized with Plot2 (http://plot2doc.micw.eu). For illustrative purposes, the genome coverage panels in Figures 2 and 3 were normalized by removing (*deduplicating*) reads based on identical alignment start sites using the SAMtools *rmdup* command. For each alignment start site, only the highest-quality read was retained for forward and reverse alignment orientations. Therefore, for the 100-bp read data shown in each coverage panel, the theoretical maximum depth is 200×.

Anellovirus contigs were assembled from the precapture sequence data using IDBA-UD (Peng et al. 2012). Contigs were aligned against the sequence database used to design the ViroCap panel using BLAST (Altschul et al. 1997) with the following parameters to detect low-similarity sequences: -G 5 -E 2 -r 1 -q -1. The percentage identity of the top HSP is reported in Supplemental Table S3.

## Data access

Data files associated with our ViroCap panel are publicly available through the https://github.com/WashU-PMG/ViroCap GitHub repository. Hosted files include 198.9 Mb of ViroCap target sequence in FASTA format, taxonomy information, corresponding RefSeq and Genome Neighbor associations, and NimbleGen's target design coverage metrics for 0- and 100-bp-offset intervals. MSS data used for ViroCap evaluation have been submitted to (with potentially identifiable human sequences removed) the NCBI BioProject database (http://www.ncbi.nlm.nih.gov/bioproject) under accession number PRJNA273884.
